# The effect of long-term danazol treatment on haematological parameters in hereditary angioedema

**DOI:** 10.1186/s13023-016-0386-2

**Published:** 2016-02-25

**Authors:** Kinga Viktória Kőhalmi, Nóra Veszeli, Zsuzsanna Zotter, Dorottya Csuka, Szabolcs Benedek, Éva Imreh, Lilian Varga, Henriette Farkas

**Affiliations:** Hungarian Angioedema Centre, 3rd Department of Internal Medicine, Semmelweis University, Kútvölgyi street 4, H-1125 Budapest, Hungary; Urology Department, Medical Centre, Hungarian Defence Forces, Budapest, Hungary; 3rd Department of Internal Medicine, Semmelweis University, Budapest, Hungary; Department of Laboratory Medicine, Semmelweis University, Budapest, Hungary

**Keywords:** Hereditary angioedema, C1-inhibitor deficiency, danazol, Prophylaxis, Haematology, Erythrocytosis, Polyglobulia

## Abstract

**Background:**

The 17-alpha-alkylated derivatives of testosterone are often used for the prevention of oedematous episodes in hereditary angioedema with C1-inhibitor deficiency (C1-INH-HAE). However, these agents can have many adverse effects, including erythrocytosis and polyglobulia. Our aim was to investigate occurrence of erythrocytosis and polyglobulia after long-term danazol prophylaxis in C1-INH-HAE.

**Methods:**

During the initial stage of our retrospective study, we explored whether C1-INH-HAE is associated with susceptibility to erythrocytosis and/or polyglobulia. In the second stage, we analyzed the haematological parameters of 39 C1-INH-HAE patients before, as well as after treatment with danazol for 1, 3, or 5 years. In the third stage, we studied the incidence of erythrocytosis and of polyglobulia after dosing with danazol for more than 5 years.

**Results:**

We did not find any significant difference between C1-INH-HAE patients not receiving danazol and healthy controls as regards the occurrence of erythrocytosis or polyglobulia. The haematological parameters did not change after treatment with danazol for 1, 3, or 5 years. Platelet count was an exception-it decreased significantly (*p* = 0.0115) versus baseline, but within the reference range. Treatment-related polyglobulia did not occur. We observed erythrocytosis in a single female patient after 1-year-and in three female patients after more than 5-year long-treatment with danazol. Erythrocytosis did not require intervention or the discontinuation of danazol therapy.

**Conclusions:**

We conclude that neither erythrocytosis, nor polyglobulia occurs more often in C1-INH-HAE patients than in healthy individuals; it can be observed only sporadically even after treatment with danazol.

## Background

Hereditary angioedema resulting from the deficiency of the C1-inhibitor (C1-INH-HAE) is a rare autosomal dominant disorder. Its characteristic feature is bradykinin-mediated angioedema, manifested by sudden episodes of subcutaneous and/or submucosal edema-formation. The management of the disease consists of acute treatment, as well as of the prevention of oedematous episodes. Currently, antifibrinolytic agents, attenuated androgens (AAs), plasma-derived C1-INH concentrate, and progestins are available for prophylaxis [1]. In C1-INH-HAE, the earliest and the most extensive experience is available with attenuated androgens (e.g., methyltestosterone, danazol, stanozolol, oxandrolone); however, the precise mode of the action of these drugs is unknown. AAs are thought to increase serum C1-INH level by stimulating hepatic synthesis, and the expression of C1-INH mRNA in peripheral blood mononuclear cells [2, 3]. Treatment with these agents reduces the frequency and severity of attacks in 94–100 % of patients; however, 5–8 % do not respond to danazol [4–6]. Even today, attenuated androgens are commonly used for the prophylactic treatment of C1-INH-HAE. However, these effective and inexpensive agents for oral use can cause a variety of adverse events [7].

The well-known side effects of AAs include the alteration of the plasma lipid profile [8], hepatotoxicity [5], virilisation [9], psychiatric and behavioural effects [10–12], the premature closure of epiphyses [13, 14], impaired glucose tolerance and insulin resistance [15, 16], as well as hypogonadism [17].

Moreover, AAs can influence haematological parameters: their stimulatory effect on erythropoiesis may result in erythrocytosis, and in the elevation of haemoglobin level [7]. Erythrocytosis and polyglobulia increase blood viscosity. As a result, the slowing of the circulation may cause dizziness, headache, visual disturbances, mental decline, as well as enhanced propensity to thromboembolism in the blood vessels of the brain, limbs, and the heart. Published experience with the haematological adverse effects of AAs has been accumulated during the danazol treatment of patients with endometriosis [18], benign breast disease [19], Fanconi anaemia [20], and idiopathic thrombocytopenic purpura (ITP) [21].

However, the findings from these studies are not fully applicable to C1-INH-HAE patients, because this disorder requires longer drug therapy (lasting for years, vs. 2–6 months in endometriosis), although with smaller doses (33–200 mg vs. 400–600 mg in endometriosis, or 50–800 mg in ITP) [21]. In the literature, erythrocytosis and polyglobulia are mentioned in two publications [4, 5], whereas polyglobulia is identified in another paper [22], as a possible adverse effect of AAs; however, long-term follow-up studies have not yet been conducted in this subject.

In view of the foregoing, we intended to explore-by implementing a follow-up study-the effects of long-term danazol therapy on the haematological parameters of C1-INH-HAE patients. We also investigated whether treatment with danazol could cause eryhtrocytosis and/or polyglobulia.

## Methods

### Study subjects

#### Patients

We conducted our prospective study in the National Angioedema Centre (Budapest, Hungary). We investigated the incidence of erythrocytosis and polyglobulia among 145 C1-INH-HAE patients, diagnosed and followed up at the Centre in compliance with the international criteria [23]. The periodic follow-up evaluation performed on these patients at least once a year includes, among others, a haematology screen. The laboratory findings are recorded in the National HAE Register; we analyzed the data accumulated between 1993 and 2015.

#### Controls

In the age- and gender-matched group of healthy controls, the haematological parameters for analysis were obtained from a blood sample drawn for the purposes of a health status screening. The demographic properties of these subjects are detailed in the Results section.

All patients gave informed consent; and the study was endorsed by the Ethical Committee of the Semmelweis University (Budapest), and it was implemented in conformity with the declaration of Helsinki.

### Complete blood count

The haematological parameters – white blood cell count (WBC), red blood cell count (RBC), haemoglobin level (Hb), haematocrit (Hct), and platelet count (PLT)-were measured with a digital analyzer (Cobas Integra 400/800, Roche, Basel, Switzerland). Erythrocytosis was defined as a RBC count >5.9 × 10^12^/L in males and >5.1 × 10^12^/L in females. The threshold values for polyglobulia were Hct >0.52 L/L in males, and >0.48 L/L in females [24].

### Study design

#### In the first stage of the study

We compared the incidence of erythrocytosis and of polyglobulia in C1-INH-HAE patients who have never been treated with danazol, and in healthy controls. We used the following two methods for this purpose:

1. We took into account the initial laboratory findings of C1-INH-HAE patients over the age of 18 years, who had never before received danazol. Seventy-six of the 145 C1-INH-HAE patients fulfilled these criteria. By analyzing RBC and Hct values, we ascertained the occurrences of erythrocytosis and of polyglobulia among these patients and among the controls.
2. In the year 2012, there were 51 individuals among the 145 C1-INH-HAE patients, who had never before received danazol. We compared the haematological parameters of these patients with those of the controls to determine the number of cases with erythrocytosis and polyglobulia in these two groups.

#### In the second stage of the study

We examined the influence of long-term danazol treatment on the haematological parameters of the patients, as well as looked for relationships among the latter and danazol dose. To this end, we compared the haematological parameters measured before the start of danazol therapy with those obtained after 1, 3, and 5 years of treatment in 39 out of 145 C1-INH-HAE patients. The haematological values were determined in the same laboratory, using the same method. Two female patients were excluded from the analysis, as they had erythrocytosis even before the start of danazol treatment.

#### In the third stage of the study

We surveyed the incidence of erythrocytosis and polyglobulia in C1-INH-HAE patients who had received danazol (50–200 mg/day) longer than 5 years. We determined the duration of danazol treatment by reviewing the database of the National HAE Registry, as well as the medical records of the patients-and then, created groups according to the duration of treatment (5–10 years, 10–15 years, 15–20 years, 20–25 years, and 25– 30 years). We ascertained the incidence of erythrocytosis and of polyglobulia by taking into account the laboratory findings obtained for the first time after the discontinuation of danazol.

### Statistical analysis

We performed the statistical analyses with the GraphPadPrism software, version 6.00 (GraphPad Software, San Diego, California, USA). The incidences of erythrocytosis and of polyglobulia were compared using Fischer’s exact test. Samples drawn before the start, as well as after 1, 3, or 5 years of danazol treatment were evaluated with paired *t*-test (Wilcoxon test), and ANOVA, by applying Spearman’s correlation during the analysis of danazol doses. A *p* < 0.05 was considered statistically significant in all analyses.

## Results

### The incidence of erythrocytosis and of polyglobulia in C1-INH-HAE patients who have never received danazol, compared with healthy controls

1. We did not find any difference (*p* > 0.05) between C1-INH-HAE patients never treated with danazol (*n* = 76; 31 males [mean age: 31.01 years, min.: 18.10 years, max.: 58.50 years]) and 45 females [mean age: 33.21 years, min.: 18.10 years, max.: 73.21 years)]) and healthy controls (*n* = 141; 57 males [mean age: 31.00 years, min.: 21.00 years, max.: 37.00 years] and 84 females [mean age: 33.00 years, min.: 22.00 years, max.: 52.00 years]), as regards the incidence of erythrocytosis and of polyglobulia (Table 1).

**Table 1:** The incidence of erythrocytosis and polyglobulia in C1-INH-HAE patients who have never been treated with danazol, vs. healthy controls

		C1-INH-HAE patients never treated with danazol (*n* = 76)	Healthy controls (*n* = 141)
Erythrocytosis	Males	0	1
	Females	1	2
No erythrocytosis	Males	31	56
	Females	44	82
Polyglobulia	Males	0	0
	Females	0	0
No polyglobulia	Males	31	57
	Females	45	84
2. Comparing the laboratory parameters obtained in the year 2012 from C1-INH-HAE patients who had never before received danazol (*n* = 51; 21 males [mean age: 37.39 years, min.: 18.10 years, max.: 63.27 years], and 30 females [mean age: 38.64 years, min.: 22.50 years, max.: 82.02 years)]) with those of age- and gender-matched healthy controls (*n* = 210; 89 males [mean age: 38.00 years, min.: 23.00 years, max.: 67.00 years] and 121 females [mean age: 38.00 years, min.: 21.00 years, max.: 59.00 years]) did not reveal any difference either in the incidence of erythrocytosis and/or polyglobulia-see Table 2.

**Table 2:** The incidence of erythrocytosis and polyglobulia (based on haematology parameters measured in 2012) in C1-INH-HAE patients who have never been treated with danazol, vs. in healthy controls

		C1-INH-HAE patients never treated with danazol (*n* = 51)	Healthy controls (*n* = 210)
Erythrocytosis	Males	0	2
	Females	1	6
No erythrocytosis	Males	21	87
	Females	29	115
Polyglobulia	Males	0	0
	Females	0	1
No polyglobulia	Males	21	89
	Females	30	120

### The effect of long-term treatment with danazol on haematological parameters

**Fig. 1:**
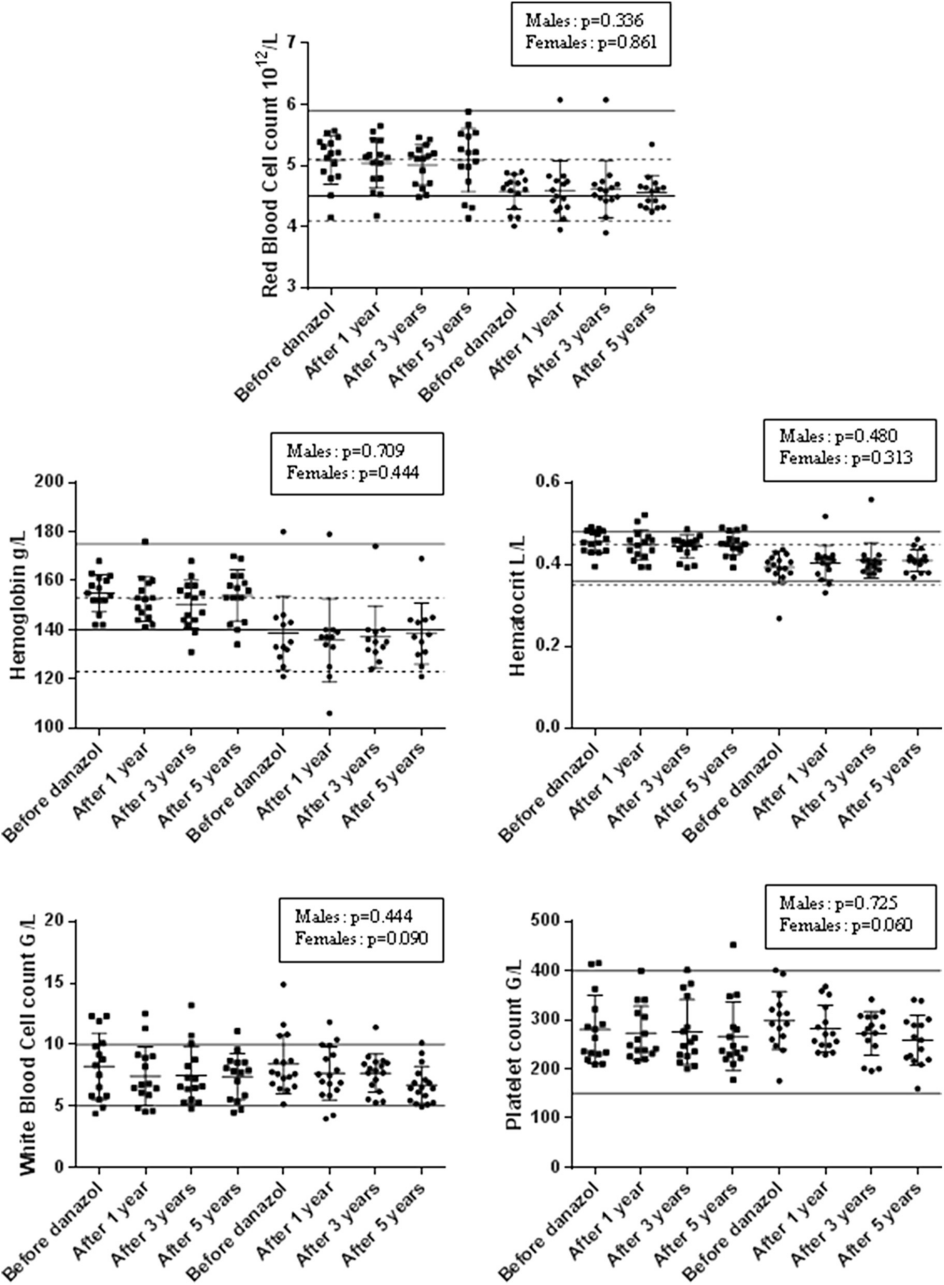
RBC, Hb, Hct, WBC, and PLT values of the 39 C1-INH-HAE patients treated with danazol at baseline and after 1, 3, and 5 years of dosing. RBC, Hb, Hct, WBC, and PLT values of the 39 C1-INH-HAE patients (18 males and 21 females) treated with danazol at baseline and after 1, 3, and 5 years of dosing (ANOVA test). Males are identified by black squares, whereas females are identified by black dots. Where different for males and females, the reference range is depicted by a continuous line for the former, and by a dotted line for the latter

We compared the haematological parameters measured before the start of danazol therapy with those obtained after 1, 3, and 5 years of treatment-in 39 out of 145 C1-INH-HAE patients (37 with HAE type I and 2 with HAE type II) – see Fig. 1. During the 5-year long follow-up, data were available from a smaller number of patients during years 3 to 5 than in the initial year. The demographic data of the patients are detailed as follows.

#### Patients receiving one-year danazol prophylaxis

Thirty-nine patients received danazol prophylaxis for a year. This group included 18 males (mean age: 37.23 years, min.: 18.00 years, max.: 66.46 years) and 21 females (mean age: 36.72 years, min.: 18.26 years, max.: 65.57 years).

#### Patients receiving three-year danazol prophylaxis

Thirty-three patients were enrolled into this group, which comprised 16 males (mean age: 35.64 years, min.: 18.00 years, max.: 45.46 years) and 17 females (mean age: 33.81 years, min.: 18.27 years, max.: 47.29 years).

#### Patients receiving five-year danazol prophylaxis

Danazol prophylaxis was administered over 5 years to 30 patients-that is, to 15 males (mean age: 35.17 years, min.: 18.00 years, max.: 45.46 years) and 15 females (mean age: 34.32 years, min.: 18.27 years, max.: 47.29 years).

Compared with baseline, we could not detect any significant changes in the haematological parameters of the male patients treated with danazol for 1, 3, or 5 years. As regards the female patients, the same applies to RBC, Hb, and Hct. However, WBC and PLT values were significantly (*p* = 0.0067, and *p* = 0.0203) lower after 5 years of danazol treatment than at baseline (Table 3). We observed RBC and Hct values beyond the upper limit of the normal range in a single patient. In this female subject (Patient #1), erythrocytosis occurred after one-year treatment with danazol, and persisted throughout the follow-up period (i.e., beyond years 3 and 5). This patient started taking danazol at the age of 44 years. As her last menses occurred at the age of 46 years, erythrocytosis cannot be interpreted as a menopausal change. The patient has never smoked in her life. The specialist consultation done in 2012 excluded the presence of any unrecognised haematological disorder (the patient was negative for the JAK-2 mutation), and recommended watchful waiting. Danazol treatment was not stopped. In consideration of the foregoing, this case of erythrocytosis could be attributed to danazol treatment.

**Table 3:** The median values of the haematological parameters of the 39 C1-INH-HAE patients taking danazol before treatment as well as after 1-, 3-, and 5-year treatment with danazol

Parameter	Gender	Reference range	Median before 1-year treatment	Median after 1-year treatment	Median before 3-year treatment	Median after 3-year treatment	Median before 5-year treatment	Median after 5-year treatment
WBC	Males	4.0–10.0 × 10^9^/L	8.54 (5.83–10.5)	6.76 (5.70–9.06)	7.90 (5.83–10.0)	6.57 (5.40–8.63)	8.30 (5.83–10.10)	7.83 (5.52–8.53)
	Females		7.60 (6.70–10.57)	7.80 (6.55–9.22)	7.60 (7.03–9.88)	7.88 (6.48–8.61)	7.60 (6.80–10.7)	6.45 (5.43–7.12)*
RBC	Males	4.50–5.90 × 10^12^/L	5.17 (4.82–5.37)	5.14 (4.79–5.35)	5.21 (4.84–5.38)	5.14 (4.70–5.29)	5.20 (4.82–5.39)	5.21 (4.74–5.52)
	Females	4.10–5.10 × 10^12^/L	4.60 (4.33–4.73)	4.61 (4.31–4.81)	4.60 (4.33–4.73)	4.55 (4.45–4.69)	4.62 (4.27–4.76)	4.60 (4.32–4.67)
Hb	Males	140–175 g/L	154 (149–161)	153 (146–160)	154 (152–161)	154 (144–158)	156 (151–161)	157 (151–162)
	Females	123–153 g/L	133 (124–142)	137 (127–140)	133 (122–141)	135 (131–139)	133 (124–142)	138 (129–144)
Hct	Males	0.36–0.48 L/L	0.46 (0.43–0.48)	0.45 (0.42–0.46)	0.46 (0.43–0.48)	0.45 (0.43–0.46)	0.45 (0.43–0.48)	0.45 (0.44–0.48)
	Females	0.35–0.45 L/L	0.39 (0.38–0.42)	0.42 (0.38–0.42)	0.39 (0.38–0.42)	0.40 (0.39–0.41)	0.39 (0.38–0.42)	0.41 (0.38–0.43)
PLT	Males	150–400 × 10^9^/L	270 (232–300)	254 (230–315)	270 (231–314)	258 (227–333)	263 (230–322)	241 (222–285)
	Females		291 (246–329)	265 (248–323)	302 (250–346)	286 (253–313)	302 (257–336)	262 (218–297)*

The median values of the haematological parameters of the 39 C1-INH-HAE patients (18 males and 21 females) taking danazol (with 25th and 75th percentiles in parentheses) before treatment as well as 1-, 3-, and 5-year treatment with danazol, as analyzed by a paired (Wilcoxon’s) *t*-test. A significance of *p* < 0.05 is indicated by an asterisk (*)

Elevated Hct was detected in the same female patient who developed erythrocytosis as well. This patient had polyglobulia after treatment with danazol for 1 and 3 years. However, polyglobulia was no longer detectable after 5 years, and hence its relation to danazol was improbable.

In males, RBC and/or Hct values beyond the reference range were not observed-neither before, nor after treatment with danazol for 1, 3, or 5 years.

We also studied whether the dose of danazol influences haematological parameters (WBC, RBC, Hb, Hct, and PLT). We subtracted the laboratory values obtained in year 5 from baseline values, and compared the results with the daily dose of danazol. After 5 years of prophylaxis, the mean daily dose of danazol was 106.5 (50–200) mg in males, and 108 (50–220) mg in females.

The Spearman’s correlation test did not reveal-either in males, or in females-any significant relationship between danazol dose and the values of haematological indices after 5 years of danazol treatment.

III. Fifty patients altogether (25 males and 25 females) received danazol for more than 5 years. The duration of treatment was 5–10 years in 12 patients (6 females and 6 males); >10–15 years in 21 patients (10 males and 11 females); >15–20 years in 10 patients (5 males and 5 females); >20–25 years in 6 patients (3 males, 3 females); and >25– 30 years in a single male patient. None of the male patients developed erythrocytosis or polyglobulia, whereas erythrocytosis occurred in three female patients; we did not observe polyglobulia even after treatment with danazol for 5 years. Of these three female patients, we described the case of Patient #1 in the foregoing. In this instance, erythrocytosis persisted 16 years after the start of danazol treatment (with a mean daily dose of 116.7 [50–200] mg). As regards the other two female patients with erythrocytosis, we monitored Patient #2 for 9 years, but did not detect any increase of RBC count-except the year 2015. It must be noted, however, that at the time of blood sampling, there were 25–30 WBCs, 2–3 RBCs with many epithelial cells and bacteria in the urinary sediment, as well as the urine culture was positive for *Streptococcus agalactiae.* This patient took danazol in an average daily dose of 58.3 (50–100) mg. We have been monitoring the laboratory parameters of Patient #3 for 20 years. During this period, we found RBC values above 5.1 × 10^12^/L only during the last 2 years. Nevertheless, this patient had additional predisposing factors for erythrocytosis (i.e., smoking and hypertension). This patient received danazol in a mean daily dose of 147.1 mg (100–300 mg) for 20 years.

## Discussion

These findings appear remarkable, as our study was the first to demonstrate the lack of any difference between C1-INH-HAE patients and healthy individuals as regards the incidence of erythrocytosis and of polyglobulia. Another important aspect of this study is that it analyzed these reactions-possible adverse effects of long-term danazol treatment-during the systematic and long-term follow-up of a larger patient population, by taking into account their medical history, age and gender, as well as the dose and duration of danazol treatment. Although both erythrocytosis and polyglobulia have been reported in C1-INH-HAE patients as adverse events associated with long-term danazol prophylaxis, we did not observe any significant increase of RBC and Hct values even after 5 years of treatment [4, 5, 22]. In particular, the RBC and Hct values of these patients remained within the reference range – with the exception of three female patients. However, the causal role of danazol in inducing erythrocytosis may be considered in only one of these three patients.

The dose of danazol did not influence haematological parameters. This is an apparent benefit from our effort to administer the lowest effective dose, which did not exceed 220 mg. Bork et al. reported similar observations: RBC increased in only two out of their 118 C1-INH-HAE patients on danazol treatment, which was not discontinued, notwithstanding the laboratory abnormalities [5]. Cicardi et al. studied 61 C1-INH-HAE patients receiving long-term danazol therapy, and found mild polyglobulia in just one male, and in one female subject [4]. However, these studies did not specify the dose and the duration of danazol treatment, or the details of the patient follow-up performed to detect erythrocytosis. Zurlo & Frank [22] published somewhat different results: they observed polyglobulia in 18 out of their 82 C1-INH-HAE patients on long-term danazol prophylaxis. It is important to note that at the outset, these patients received danazol in a much larger, 600 mg/day dose, which was tapered to the lowest effective level only later. The highest observed Hb and Hct values were 182 g/L, and 0.52, respectively. Zurlo & Frank diagnosed thrombocytosis in 15 C1-INH-HAE patients, but none of their subjects with polyglobulia or thrombocytosis suffered thrombotic events [22]. Danazol has been observed to stimulate thrombopoiesis-this is why it is used for the treatment of ITP [21]. Considering this along with our findings, we consider the significant reduction of platelet count in female C1-INH-HAE patients after 5 years of danazol treatment particularly intriguing and remarkable.

## Conclusion

In summary of our results, we can conclude that treatment with the lowest effective (33–220 mg/day) doses of danazol-an agent used for the treatment of C1-INH-HAE in clinical practice for nearly four decades-did not induce haematological abnormalities that would require the discontinuation of dosing, even after long-term use. Because of the sporadic occurrence of erythrocytosis observed in our study, patients on long-term danazol therapy should undergo follow-up evaluation once a year at the least. On these occasions, it is recommended to check haematological parameters in addition to the appraisal of hepatic and renal function, as well as of the serum lipid profile, and to performing abdominal ultrasound imaging. This practice affords early recognition of the adverse effects of danazol-and hence, it makes it possible to prevent consecutive disorders and to adjust the therapy as necessary.

## Abbreviations

AA: attenuated androgen
C1-INH: c1-inhibitor
C1-INH-HAE: hereditary angioedema with C1-INH deficiency
HAE: hereditary angioedema
Hb: haemoglobin
Hct: haematocrit
ITP: idiopathic thrombocytopenic purpura
PLT: platelet count
RBC: red blood cell count
WBC: white blood cell count
